# Adipose–Joint Crosstalk in Obesity–Induced Osteoarthritis: Mechanisms, Biomarkers, and Translational Therapeutic Opportunities

**DOI:** 10.1007/s11926-026-01229-9

**Published:** 2026-08-08

**Authors:** Wei Hang, Kathy Triantafilou, You Zhou

**Affiliations:** 1 Department of Infection and Immunity, School of Medicine, Cardiff University, Cardiff, CF14 4XN UK; 2 Systems Immunity Research Institute, Cardiff University, Cardiff, CF14 4XN UK

**Keywords:** Osteoarthritis, Obesity, Inflammation, Metabolism, Single-cell, Therapeutic strategies

## Abstract

**Purpose of Review:**

Obesity-induced osteoarthritis (OA) is emerging as a distinct metabolic phenotype fundamentally different from mechanically driven OA. Current therapies largely target downstream joint damage and fail to address underlying adipose–joint pathological interactions. This review synthesises recent advances in understanding how systemic metabolic dysfunction, adipose tissue inflammation, and cellular heterogeneity revealed by single-cell analyses drive OA pathogenesis, and highlights emerging strategies targeting metabolic dysfunction and chronic inflammation.

**Recent Findings:**

We critically summarised human and experimental studies investigating the role of dysfunctional adipose depots, including visceral, subcutaneous, and infrapatellar fat, in joint degeneration. Mechanistic findings on adipokine signalling, immune activation, and metabolic stress were integrated with recent single-cell insights and therapeutic interventions addressing systemic metabolism and inflammation. Obesity promotes adipose tissue hypertrophy, hypoxia, and inflammatory adipokine secretion, which remodel systemic metabolism and alter the function of joint-resident cells. Crosstalk between adipocytes, macrophages, fibroblasts, and chondrocytes sustains chronic low-grade inflammation, oxidative stress, and extracellular matrix degradation. Single-cell analyses have revealed fibroblast and macrophage subsets that mediate depot-specific inflammatory circuits. Recent findings also point to metabolic modulators (GLP-1 receptor agonists), anti-adipokine agents, and regenerative strategies as promising interventions to modify disease progression.

**Summary:**

Obesity-induced OA arises from multi-level metabolic and inflammatory crosstalk between adipose and joint tissues. Deciphering adipose–joint crosstalk provides a foundation for precision therapies that address the upstream metabolic and inflammatory drivers of joint degeneration.

## Introduction

Osteoarthritis (OA) is the most prevalent musculoskeletal disease, affecting over 7% of the global population and ranking among the leading causes of disability worldwide [1]. The convergence of obesity and OA epidemics poses an escalating public health challenge: by 2035, over 4 billion people will be overweight [2], with obesity conferring a 2.6–6.8 fold increased OA risk [3]. As populations age and obesity rates climb, these intertwined epidemics amplify one another, driving unsustainable disability and healthcare costs.

Traditionally, OA has been attributed to mechanical overload and age-related degeneration [4]. However, emerging evidence defines obesity-induced OA as a distinct metabolic phenotype, with features that transcend simple biomechanical stress [5]. Unlike age-related OA, this metabolic phenotype affects both weight-bearing (knee, hip) and non-weight-bearing (hand, temporomandibular) joints, implicating metabolic drivers beyond mechanical load. Dysfunctional adipose depots secrete pro-inflammatory cytokines and adipokines that establish chronic low-grade inflammation [6]. Metabolic dysfunction precedes [7] and drives structural joint changes, rather than resulting from mechanical damage. Critically, obesity-induced OA shows potential for prevention and even reversal through targeted metabolic interventions [8]. These defining features suggest that understanding the molecular and cellular mechanisms underlying this disease is essential for developing mechanism-based therapeutic approaches.

Recent reviews have examined various aspects of the obesity-OA relationship, including inflammatory mediators and metabolic dysregulation [9, 10]. Our synthesis advances this field by integrating depot-specific adipose tissue dysfunction with convergent molecular signalling networks and detailed cellular crosstalk, bridging tissue-level pathology with cellular heterogeneity that previous work has addressed separately. We extensively incorporate single-cell RNA sequencing and spatial transcriptomics findings to characterise the functional diversity of synovial macrophages, fibroblasts, and chondrocytes within the obesity-affected joint microenvironment [11–13]. Beyond mechanistic insights, we organize therapeutic interventions within a temporal framework spanning early metabolic dysfunction through advanced structural disease, providing stage-appropriate treatment strategies [14]. Critically, we establish obesity-induced OA as a distinct metabolic disease phenotype that affects joints, and where targeted metabolic interventions demonstrate potential for disease prevention and reversal. This comprehensive, mechanistically grounded synthesis connects molecular pathways with cellular dynamics and clinical translation, advancing both understanding and treatment of obesity-induced OA.

This review examines molecular mechanisms underlying obesity-induced OA, delineates the intercellular communication networks between adipose tissue and joints, and discusses therapeutic strategies targeting upstream metabolic dysfunction.

## Pathophysiological Mechanisms Linking Obesity to Osteoarthritis

**Fig. 1:**
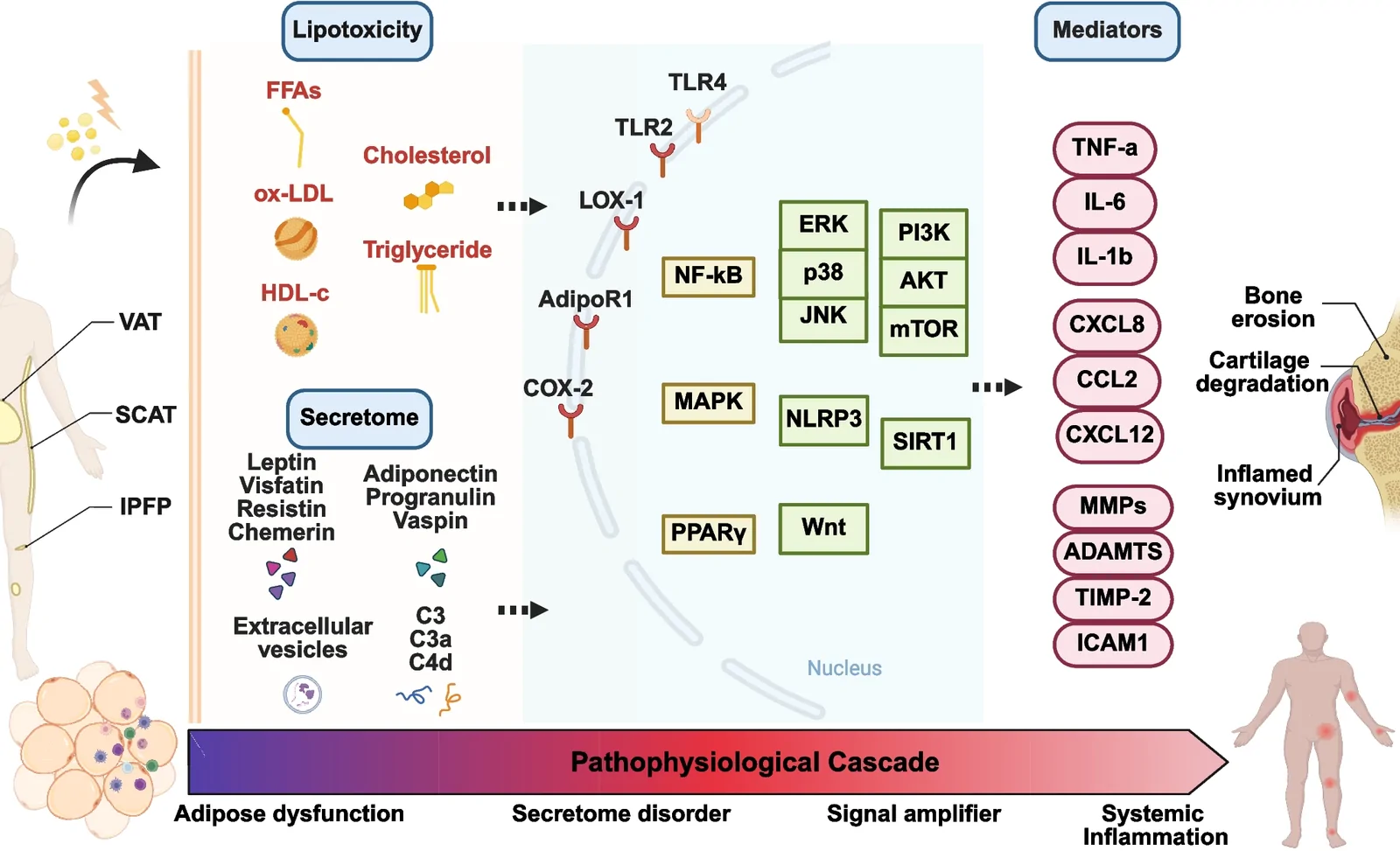
Molecular mechanisms of obesity-induced OA. The diagram illustrates the cascade from adipose dysfunction to systemic inflammation in obesity-induced osteoarthritis (OA). Adipose tissue dysfunction (left panel) encompasses three major fat depots: visceral adipose tissue (VAT), subcutaneous adipose tissue (SCAT), and infrapatellar fat pad (IPFP). These depots show altered lipid profiles, elevated free fatty acids (FFAs), oxidized low-density lipoprotein (ox-LDL), and triglycerides, and reduced high-density lipoprotein (HDL-cholesterol), alongside disrupted adipokine secretion (leptin, adiponectin, visfatin, resistin, chemerin, progranulin, and vaspin) and release of extracellular vesicles (EVs), microRNAs (miRNAs), damage-associated molecular patterns (DAMPs), and lipotoxic mediators. Signal amplification occurs through pattern recognition receptors, Toll-like receptor 2 (TLR2), TLR4, lectin-like oxidized LDL receptor 1 (LOX-1), adiponectin receptor 1 (AdipoR1), and cyclooxygenase-2 (COX-2), activating inflammatory pathways including nuclear factor kappa B (NF-κB), mitogen-activated protein kinase (MAPK), including c-Jun N-terminal kinase (JNK), extracellular signal-regulated kinase (ERK), phosphoinositide 3-kinase (PI3K), protein kinase B (AKT), and mechanistic target of rapamycin (mTOR), peroxisome proliferator-activated receptor gamma (PPARγ), NLR family pyrin domain-containing 3 (NLRP3) inflammasome, sirtuin 1 (SIRT1), and Wingless/Int-1 (Wnt)/β-catenin signalling. Systemic inflammation (right panel) is driven by pro-inflammatory mediators, tumour necrosis factor alpha (TNF-α), interleukin-6 (IL-6), IL-1β, and prostaglandin E2 (PGE2), and chemokines, C-X-C motif chemokine ligand 8 (CXCL8), C–C motif chemokine ligand 2 (CCL2), CXCL12, resulting in matrix degradation through matrix metalloproteinases (MMPs), a disintegrin and metalloproteinase with thrombospondin motifs (ADAMTS), and tissue inhibitor of metalloproteinases 2 (TIMP-2). (Created in BioRender. Z, Y. (2026) https://BioRender.com/aotyf3k)

As illustrated in Fig. 1, obesity-induced OA is driven by a complex interplay among dysfunctional adipose depots, systemic metabolic perturbations, and joint tissue responses. The pathophysiological cascade begins with adipose tissue dysfunction, progresses through lipid and secretome dysregulation, and converges on integrated molecular signalling networks that drive joint destruction.

### Adipose Tissue Dysfunction as Primary Pathological Driver

Obesity-induced OA originates from dysfunction of anatomically distinct adipose tissue depots that disrupt both local and systemic homeostasis.

Visceral adipose tissue (VAT) serves as the primary systemic inflammatory engine. In humans with obesity, ex vivo secretome profiling of VAT demonstrates increased secretion of pro-inflammatory cytokines (TNF-α, IL-6, IL-1β) and pro-catabolic (elevated free fatty acids and oxidized lipoproteins) signalling that directly reach joint tissues via circulation [15]. In patients with obesity, histopathological analysis of visceral adipose tissue reveals progressive morphological and inflammatory changes associated with adipocyte insulin resistance, which promotes enhanced lipolysis and inflammatory mediator secretion [16].

Subcutaneous adipose tissue (SCAT) contributes through dual mechanical and metabolic mechanisms. In mice, SCAT dysfunction leads to ectopic lipid deposition within joints, inducing lipotoxic effects on chondrocytes and synoviocytes [17].

The infrapatellar fat pad (IPFP) provides the most direct adipose-joint link. In diet-induced obese mice, IPFP undergoes fibrotic remodelling with crown-like macrophage structures surrounding dying adipocytes [18]. Single-cell and spatial transcriptomic analyses reveal depot-specific adipocytes that initiate immune-stromal interactions, amplifying local inflammation [19]. In a high-fat die-fed mice model, IPFP adipocyte expansion and inflammatory mediator upregulation create a local cytokine source that directly affects adjacent joint structures [20].

Beyond these, emerging adipose compartments like intramuscular adipose tissue (IMAT) and bone marrow adipose tissue (BMAT) also contribute to the complexity, though their roles require further investigation.

### Lipid Dysregulation

Lipid metabolic disruptions create the biochemical conditions for subsequent inflammatory cascades. Free fatty acids (FFAs) directly activate Toll-like receptors (TLR2/4) on tissue-resident macrophages infiltrating hypertrophic adipose tissue, triggering nuclear factor κB (NF-κB) signalling cascades [21]. Plasma levels of oxidized low-density lipoprotein (ox-LDL) are markedly elevated in obese individuals, whereas levels of protective high-density lipoprotein cholesterol (HDL-c) show a concurrent decline. The activated lectin-like ox-LDL receptor-1 (LOX-1) serves as a key receptor mediating ox-LDL metabolic effects, with human studies in inflammatory arthritis showing subsequent upregulation of MMP-1/3/13 and pro-inflammatory cytokines IL-1β, IL-6, and TNF-α [22]. Simultaneously, reduced HDL removes critical protective barriers. Mice with low-functional HDL show cartilage fibrillation, and increased MMP-2/9/13 expression with reduced collagen type II inducing cartilage degradation [23]. In a prospective cohort of asymptomatic middle-aged women, elevated cholesterol and triglyceride levels correlate with bone marrow lesion occurrence, contributing to cartilage loss progression and pain [24]. Collectively, lipid storage dysfunction leads to ectopic fat deposition in joint tissues, causing direct lipotoxic cellular damage.

### Secretome Alterations

The pathophysiological effects of obesity extend to complex metabolic stress through alterations in the secretome profile. These changes encompass adipokines, lipokines, extracellular vesicles, and complement components (Fig. 0.1).

Leptin is one of the most studied mediators of obesity-related joint destruction in patients with obesity, exerting deleterious effects across multiple joint tissues including IPFP, synovium, articular cartilage, and subchondral bone [25]. In human osteoarthritic cartilage explants, elevated leptin concentrations upregulate pro-inflammatory mediators (PGE2, IL-6, IL-8) while increasing matrix-degrading enzymes such as matrix metalloproteinases (MMP-1/3/13, ADAMTS-4/5/9) through NF-κB signalling [26].

Adiponectin exhibits complex, context-dependent effects, acting as a dual-function mediator. In human osteoarthritic cartilage, adiponectin promotes cartilage degradation by increasing MMP-1/3/13 and inducible nitric oxide synthase via AMPK signalling [27].It also induces ICAM-1 expression in human synovial fibroblasts promoting monocyte adhesion to synovial fibroblasts [28], and promotes IL-6 expression via NF-κB signalling [29]. Adiponectin activates NF-κB on the MMP-3 promoter through AdipoR1 promoting cartilage breakdown in human chondrocytes [30]. Conversely, adiponectin shows protective effects in human osteoarthritic chondrocytes and synovial tissue by increasing TIMP-2 [31].

Resistin, visfatin, and chemerin form an inflammatory trio with elevated levels in obesity that correlate positively with OA severity. Resistin promotes COX-2 expression through AMPK and SIRT1 dysregulation in human osteoarthritic chondrocytes [32]. Visfatin stimulates MMP-3, CCL2, and CXCL8 in synovial fibroblasts [33]. Chemerin identified in human inflammatory fluids promotes immune cell recruitment to joint tissues through calcium mobilization and chemotaxis [34]. Additional protective adipokines include progranulin, which reduces TNF-α-induced cartilage-degrading enzymes [35]. Vaspin reverses IL-1β effects and promotes cholesterol efflux in rats [36].

Extracellular vesicles carrying microRNA cargo represent advanced intercellular communication mechanisms. Exosomes derived from healthy chondrocytes can restore mitochondrial function and promote anti-inflammatory macrophage polarization [37]. In human synovial fluid, complement system activation is elevated in patients with OA, particularly C3 and activation peptides (C3a, C4d) compared to healthy knees [38].

### Molecular Signalling Convergence in Joint Tissues

Diverse secretome alterations in obesity-induced OA operate as highly integrated networks that create self-reinforcing amplification mechanisms manifesting as cartilage degradation, synovial inflammation, and subchondral bone remodeling.

Core inflammatory signalling pathways receive convergent inputs from multiple obesity-derived signals. NF-κB receives convergent inputs from TLRs detecting danger signals, cytokine receptors responding to adipokines, and damage-associated molecular patterns (DAMPs) released from stressed tissues [39]. Mitogen-activated protein kinase (MAPK) cascade, particularly ERK, p38 MAPK, and JNK pathways, amplifies inflammatory signals and coordinates MMP production [40]. Peroxisome proliferator-activated receptor γ (PPARγ) signalling functions as a critical counter-regulatory mechanism. In animal OA model, activated PPARγ reduces the production of OA mediators (MMPs, ADAMTS-5, TNF-α, PGE2, IL-1β, IL-6) and inhibits key signalling pathways (ERK1/2 MAPK, p38, AP-1, NF-κB) [41, 42]. In contrast, PPARγ deficiency reduces chondrocyte differentiation and proliferation while increasing catabolic activity, accelerating cartilage destruction in cartilage-specific PPARγ knockout mice [43].

Cellular stress response pathways determine cell fate in obesity-exposed joint tissues. The PI3K/AKT/mTOR axis has been implicated in cellular metabolism, autophagy, and survival responses in joint tissues [44]. The NLRP3 inflammasome represents a critical driver where obesity-derived cellular stress triggers caspase-1 activation, leading to IL-1β and IL-18 maturation and pyroptotic cell death [45].

Specialized molecular recognition systems in joint tissue can detect and respond to obesity-associated pathogenic signals. In a high-fat diet-fed mice model, TLR2/4 can be stimulated by FFAs, leading to downstream JNK signalling and macrophage activation within joint tissues [21].

## Adipose-Joint Crosstalk Networks

### Cellular Communication Networks Dysregulation

**Fig. 2:**
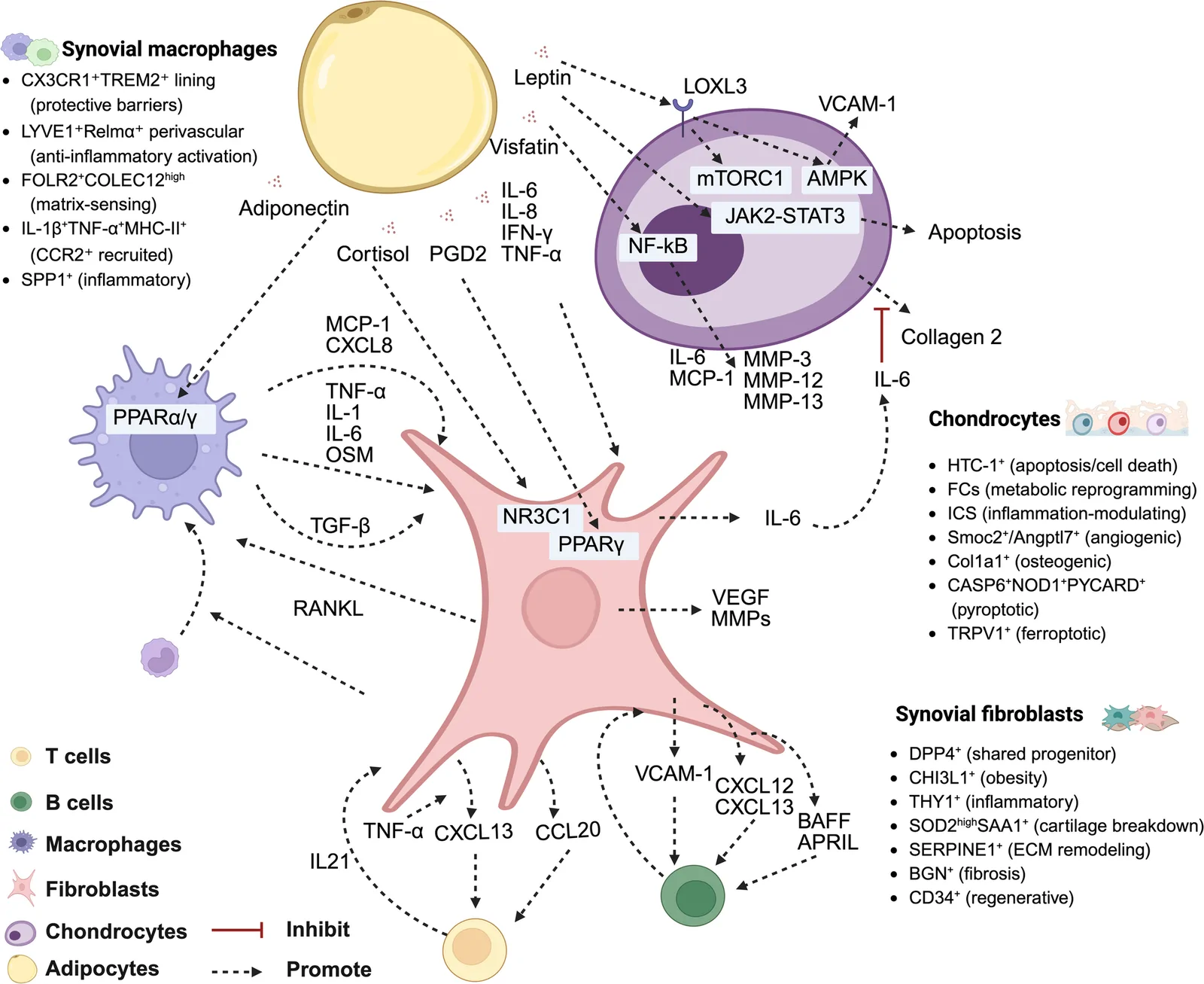
Cellular crosstalk networks in obesity-induced OA joint microenvironment. The schematic illustrates intercellular communication networks within the obesity-affected joint, with bidirectional signalling between synovial macrophages, synovial fibroblasts, chondrocytes, T cells, and B cells. Macrophages receive adipokine signals from adipose tissue, adiponectin, leptin, visfatin, cortisol, prostaglandin D2 (PGD2), TNF-α, IL-6, IL-8, and interferon gamma (IFN-γ), with polarisation modulated through PPARγ. Activated macrophages secrete pro-inflammatory mediators, TNF-α, IL-6, IL-1β, TLR ligands, oncostatin M (OSM), and chemokines, monocyte chemoattractant protein-1 (MCP-1), CXCL8, recruiting and activating synovial fibroblasts. Fibroblasts, expressing nuclear receptor subfamily 3 group C member 1 (NR3C1; glucocorticoid receptor) and PPARγ, amplify inflammation through IL-6, vascular endothelial growth factor (VEGF), and MMP production, and communicate with lymphocytes via vascular cell adhesion molecule 1 (VCAM-1), C-X-C motif chemokine ligand 12 (CXCL12), CXCL13, B-cell activating factor (BAFF), and a proliferation-inducing ligand (APRIL). Lymphocytes interact through IL-21, TNF-α, CXCL13, and C–C motif chemokine ligand 20 (CCL20). Chondrocytes respond with increased IL-6, MMP-3, MMP-12, MMP-13, and collagen 2 degradation under metabolic stress through mTOR complex 1 (mTORC1), AMP-activated protein kinase (AMPK), Janus kinase 2/signal transducer and activator of transcription 3 (JAK2-STAT3), and NF-κB. Solid lines indicate inhibitory interactions; dashed lines indicate promotional effects. Macrophage subsets: C-X3-C motif chemokine receptor 1 (CX3CR1) + triggering receptor expressed on myeloid cells 2 (TREM2) + lining, folate receptor beta (FOLR2) + CD206high matrix-sensing, C–C motif chemokine receptor 2 (CCR2) + recruited, and secreted phosphoprotein 1 (SPP1) + inflammatory. Fibroblast subsets: dipeptidyl peptidase 4 (DPP4) + shared progenitor, CHI3L1 + (obesity), thymus cell antigen 1 (THY-1) + inflammatory, superoxide dismutase 2 (SOD2)high serum amyloid A1 (SAA1) + cartilage breakdown, serpin family E member 1 (SERPINE1) + extracellular matrix (ECM) remodelling, biglycan (BGN) + fibrosis, and cluster of differentiation 34 (CD34) + regenerative. Chondrocyte subsets: apoptotic, metabolically stressed, inflammatory, osteogenic (collagen type 1 alpha 1 [Col1a1] +), pyroptotic (caspase 6 [CASP6] +), and ferroptotic subtypes. (Created in BioRender. Z, Y. (2026) https://BioRender.com/9qamm18)

Evidence suggests obesity can shift adipose tissue toward a dysfunctional, inflammatory state that sends signals to joint tissues. As illustrated in Fig. 2, dysfunctional adipose tissue initiates cascading inflammatory networks that converge on the joint microenvironment [46]. The pathological intercellular communication involves dysfunctional adipocytes, multiple immune cell populations (macrophages, T cells, B cells), synovial fibroblasts, and chondrocytes.

Adipose-derived signals directly target joint cells to initiate pathology. In both rat and human OA cartilage samples, leptin stimulates lysyl oxidase-like 3 (LOXL3) expression in chondrocytes, triggering apoptosis through mTORC1 activation [47]. Leptin also induces VCAM-1 expression via AMPK and JAK2/PI3K signalling pathways, promoting leukocyte infiltration and contributing to cartilage degradation in human primary chondrocytes [48]. Visfatin accelerates cartilage matrix degradation by inducing MMPs (MMP-3/12/13) and inflammatory mediators (IL-6, MCP-1) through NF-κB activation [49, 50]. Adipocyte-derived mediators (IL-6, IL-8, IFN-γ, TNF-α) and elevated leptin directly modulate synovial fibroblast function in patients with OA [51].

In studies across mouse models and human synovial tissue, adipocyte-derived cortisol activates glucocorticoid receptor (NR3C1) signalling in fibroblasts, maintaining a lipid-metabolic phenotype and preventing excessive matrix remodelling [52]. PPARγ agonist precursor PGD2 induces synovial fibroblast differentiation into adipocytes [53]. Similarly, PPARγ ligand troglitazone enables synovial fibroblast differentiation into adipocyte-like cells in a human fibroblast-like synovial cells model [54]. This plasticity suggests that synovial fibroblasts may contribute to ectopic adipose tissue formation within joints, potentially amplifying local adipokine production.

Within the joint, macrophage-fibroblast interactions create additional pathological amplification circuits. Evidence from inflammatory arthritis models shows that activated synovial fibroblasts recruit peripheral blood monocytes through chemokine secretion and promote their differentiation into synovial macrophages [55]. These newly recruited macrophages produce inflammatory cytokines (TNF, IL-1β, IL-6) and chemokines (CCL2, CXCL8) [56]. In mouse arthritis models, inflammatory macrophages secrete mediators (OSM), which enhance fibroblast pathogenic activation and invasiveness [57]. In response, synovial fibroblasts upregulate RANKL secretion to promote macrophage-derived osteoclast differentiation, contributing to subchondral bone remodelling [57]. In obesity, joint hypoxia exacerbates this crosstalk by enhancing macrophage-derived IL-1, TNF-α, and TGF-β, which activate fibroblasts to secrete VEGF and matrix metalloproteinases [58], promoting angiogenesis and matrix degradation.

Chondrocyte-synovial fibroblast interactions significantly influence OA progression through bidirectional inflammatory signalling. Leptin stimulates pathological crosstalk between chondrocytes and synovial fibroblasts derived from patients with OA, triggering additional IL-6 secretion from fibroblasts [59]. The IL-6 secreted by synovial fibroblasts subsequently compromises cartilage matrix integrity by inhibiting type II collagen production in human articular chondrocytes through downregulation of Sp1/Sp3 transcription factors [60].

Synovial fibroblasts orchestrate immune cell recruitment through coordinated mechanisms, established principally in rheumatoid arthritis that are increasingly recognised in OA synovium. They secrete chemokines (CXCL12, CXCL13) and express adhesion molecules (VCAM1) to recruit B cells [61], then support B cell survival through BAFF and APRIL secretion via TLR3 activation [62]. TNF-α stimulates synovial cells to produce CXCL13, recruiting T cells and amplifying inflammation [63]. In mice and human studies, synovial fibroblasts secrete CCL20 to accelerate Th17 cell recruitment [64]. IL-21 produced by Th17 subsequently promotes synovial fibroblast proliferation and activation [65].

Understanding these integrated cellular communication networks reveals therapeutic targets for disrupting the obesity-OA axis, with strategies interrupting specific circuits offering disease-modifying potential.

### Single-Cell Insights Into Cellular Heterogeneity

Advanced single-cell RNA sequencing technologies reveal distinct cellular subpopulations within OA joints and their responses to obesity-related metabolic stress, providing unprecedented resolution of how obesity reshapes cellular diversity.

Synovial macrophage subpopulations have been identified based on marker expression: transitional CX3CR1⁺TREM2⁺ lining macrophages that form protective barriers and maintain homeostasis [66], LYVE1⁺Relmα⁺ perivascular macrophages located in sub-lining regions expressing CD206 and CD163, supporting M2 alternative activation [66]. MHCII⁺CSF1R⁺ interstitial precursors provide local replenishment of tissue-resident populations [67]. Recent single-cell atlases have expanded this classification to include tissue-resident macrophage subsets with proposed matrix-sensing (FOLR2^+^COLEC12^high^) and iron-recycling (LYVE1^+^SLC40A1^+^) activities [68]. In a mouse post-traumatic OA model, synovial macrophage homeostasis was disrupted, promoting recruitment of circulating CCR2⁺ monocytes that differentiate into inflammatory macrophages expressing IL-1β, TNF-α, and MHC Class II [69]. Advanced single-cell studies have identified SPP1^+^ macrophages as key inflammatory subsets that accumulate in synovial fluid, exhibiting pronounced communication with CXCL13^+^CD4^+^ T cells and driving synovial inflammation [70].

Synovial fibroblast populations demonstrate distinct molecular endotypes with specialized pathogenic functions altered by obesity-induced metabolic stress. In obesity-induced OA, synovial fibroblasts segregate into functionally distinct subpopulations defined by obesity-specific molecular signatures, including ‘activated fibroblasts’ and ‘immune cell recruiters’ that drive synovitis [71]. The ‘immune cell recruiters’ subset specifically expresses high levels of CHI3L1, mediating inflammation in patients with obesity and OA, while patients with normal-weight and OA show elevated INHBA expression representing distinct inflammatory molecular endotypes [72]. These subpopulations exhibit spatial organization marked by differential expression of transcriptional regulators MYC and FOS, with FOS predominating in patients with obesity and regulating joint-destructive pathways [72]. Additional fibroblast diversity includes THY1^+^ inflammatory fibroblasts and THY1^−^ destructive fibroblasts [73]. Recent studies have also identified specialized subsets for cartilage breakdown (SOD2^high^ SAA1^+^ fibroblasts) versus extracellular matrix remodelling (SERPINE1^+^ fibroblasts) [68].

Cross-tissue analysis has revealed that synovium and infrapatellar fat pad share common mesenchymal progenitors, with DPP4^+^ mesenchymal cells functioning as a common progenitor for IPFP adipocytes and synovial lining layer fibroblasts, suggesting these tissues represent an integrated functional unit [74]. OA induces a profibrotic and inflammatory phenotype in mesenchymal lineage cells, with BGN^+^ intermediate fibroblasts driving tissue fibrosis [74]. Meanwhile, CD34^+^ synovial fibroblast subsets retain osteoblastic and chondrogenic potential, indicating dual destructive and regenerative capacities within the fibroblast population [75].

Advanced single-cell transcriptomic atlases have further identified hypertrophic chondrocyte (HTC-1) specifically expressing genes associated with apoptosis and programmed cell death [76]. Fibrocartilage chondrocytes exhibit fundamental metabolic reprogramming in obesity-induced OA, showing increased glycolysis and impaired proteoglycan synthesis [77]. Novel mouse subpopulations include Smoc2^+^ angiogenic chondrocytes, Angptl7^+^ angiogenic chondrocytes that promote angiogenesis in subchondral bone through FGF2-FGFR2 signalling, and Col1a1^+^ osteogenic chondrocytes [78]. Recent studies have also identified pyroptosis-related chondrocyte signatures with CASP6, NOD1, and PYCARD as prognostic factors [79]. Ferroptotic chondrocyte clusters expressing TRPV1 as an anti-ferroptotic target, revealing novel cell death pathways contributing to cartilage degeneration [80].

The cellular heterogeneity revealed by single-cell studies necessitates personalized therapeutic approaches. Patient stratification should incorporate multi-dimensional profiling including adipokine signatures, inflammatory markers, and metabolic parameters. Patient-derived synovial fluid cells can be analysed to determine individual cellular composition profiles, including macrophage polarization states, fibroblast subpopulations, and chondrocyte functional status.

### Temporal Joint Progression Model

Understanding obesity-induced OA progression through distinct phases provides essential insights for developing phase-appropriate therapeutic strategies, with early detection and intervention critical for preventing irreversible joint damage.

During the early phase of metabolic dysfunction, adipocyte hypertrophy initiates secretory profile alterations resulting in enhanced pro-inflammatory adipokine secretion (leptin, resistin, visfatin) while reducing protective factors like adiponectin. In human synovial tissue, early OA showed early inflammatory mediator elevation with initial macrophage infiltration that intensifies with histological OA grade progression [81]. This phase represents the optimal therapeutic window where targeted metabolic interventions can prevent progression.

The intermediate phase establishes self-perpetuating inflammatory cascades fundamentally altering joint tissues. IL-6 emerges as a critical regulator promoting pathogenic Th17 development through IL-6/IL-23-activated STAT3 signalling. In human OA chondrocytes in vitro, leptin regulates MMP-13 and TIMP-2 expression in a BMI-dependent manner [82], with development of hypertrophic and senescent phenotypes characterised by increased reactive oxygen species and oxidative stress [83]. Synovial fibroblasts undergo metabolic changes demonstrating greater basal lactate secretion and aerobic glycolysis compared to lean controls [84]. This phase requires multi-target interventions addressing both inflammatory cascades and metabolic dysfunction.

In the advanced phase, irreversible tissue destruction occurs with complete proteoglycan depletion, progressive subchondral bone remodelling with sclerosis and osteophyte formation. In an obesity-associated OA mouse model, enhanced M1-polarized macrophages demonstrate decreased GAS6 secretion, resulting in impaired efferocytosis [85]. Synovial fibrosis develops with loss of normal joint architecture, perpetuating destruction and preventing endogenous repair. Regenerative capacity becomes severely compromised, with synovium-derived mesenchymal stem cells losing chondrogenic differentiation potential [86]. This phase requires comprehensive disease-modifying strategies potentially including tissue engineering and surgical interventions combined with metabolic management.

### Characterising the Phenotype in Patients with Obesity-Induced OA

**Fig. 3:**
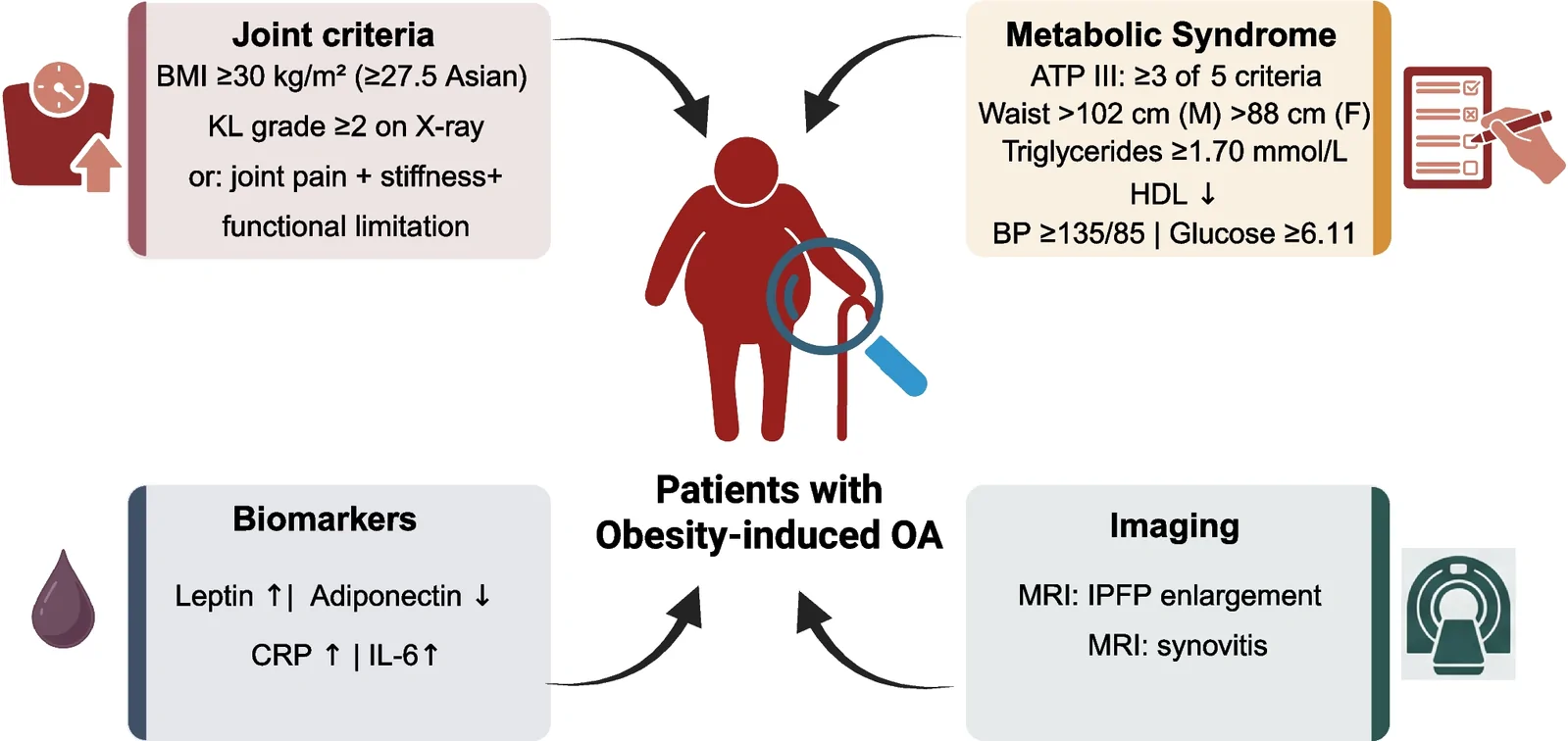
Criteria for Identifying Patients with Obesity-Induced OA. The diagram summarises the clinical, metabolic, biomarker, and imaging features used to identify the obesity-induced OA phenotype. Four criteria categories surround the central patient label. Joint Criteria: body mass index (BMI) ≥ 30 kg/m^2^ (≥ 27.5 kg/m.^2^ in Asian populations) with symptomatic OA confirmed by Kellgren-Lawrence (KL) grade ≥ 2 on X-ray, or persistent joint pain, stiffness, and functional limitation. Metabolic Syndrome: co-existing metabolic syndrome defined by Adult Treatment Panel III (ATP III) criteria: waist circumference > 102 cm (men) or > 88 cm (women), triglycerides ≥ 1.70 mmol/L, reduced high-density lipoprotein (HDL-cholesterol), blood pressure (BP) ≥ 135/85 mmHg, or fasting glucose ≥ 6.11 mmol/L. Biomarkers: raised serum leptin, reduced adiponectin, raised C-reactive protein (CRP), and raised IL-6. Imaging: infrapatellar fat pad (IPFP) enlargement or synovitis on magnetic resonance imaging (MRI). (Created in BioRender. Z, Y. (2026) https://BioRender.com/qni0xiw)

The evidence in Sections "Pathophysiological Mechanisms Linking Obesity to Osteoarthritis" and "Adipose-Joint Crosstalk Networks" supports identifying obesity-induced OA as clinically distinct from mechanically driven OA, with metabolic and inflammatory factors as the main drivers. In practice, this applies to patients with obesity (BMI ≥ 30 kg/m^2^; ≥ 27.5 kg/m^2^ in Asian populations [2]) and symptomatic OA confirmed by X-ray (Kellgren–Lawrence grade ≥ 2 [87]) or clinical criteria (persistent joint pain, stiffness, and functional limitation), as shown in Fig. 3. Critically, fat tissue signalling rather than mechanical loading appears to be the main driver of joint damage in this group [46].

Beyond BMI and joint status, this phenotype is also defined by metabolic dysfunction. Co-existing metabolic syndrome, using ATP III criteria [6], defined as three or more of: waist circumference > 102 cm (men) or > 88 cm (women), fasting triglycerides ≥ 1.70 mmol/L, reduced HDL-cholesterol, blood pressure ≥ 135/85 mmHg, or fasting glucose ≥ 6.11 mmol/L — identifies patients in whom systemic metabolic burden, rather than joint loading, is likely driving the disease. Raised serum leptin and reduced adiponectin, each linked to OA severity and progression [25, 26], reflect the adipokine shift described in Section "Secretome alterations". Raised CRP and IL-6 provide further evidence for a fat-driven inflammatory process [10]. Where imaging is available, infrapatellar fat pad enlargement or synovitis on MRI can confirm local fat-joint involvement [88]. Together, these features set obesity-induced OA apart from lean or post-traumatic OA, and are consistent with obesity-specific molecular profiles found in recent single-cell studies [72].

Identifying this patient group carries clear treatment consequences. Weight loss through diet and exercise slowed cartilage breakdown in high-BMI patients [8], and the STEP trial showed that semaglutide reduced both structural damage and symptoms specifically in patients with obesity and knee OA [89]. Metabolic testing can therefore help guide prognosis and treatment choice. No firm consensus yet exists on exact cut-off values for these criteria; the figures above reflect current evidence and should be used as practical clinical guides rather than strict thresholds.

## Therapeutic Implications and Emerging Targets

**Fig. 4:**
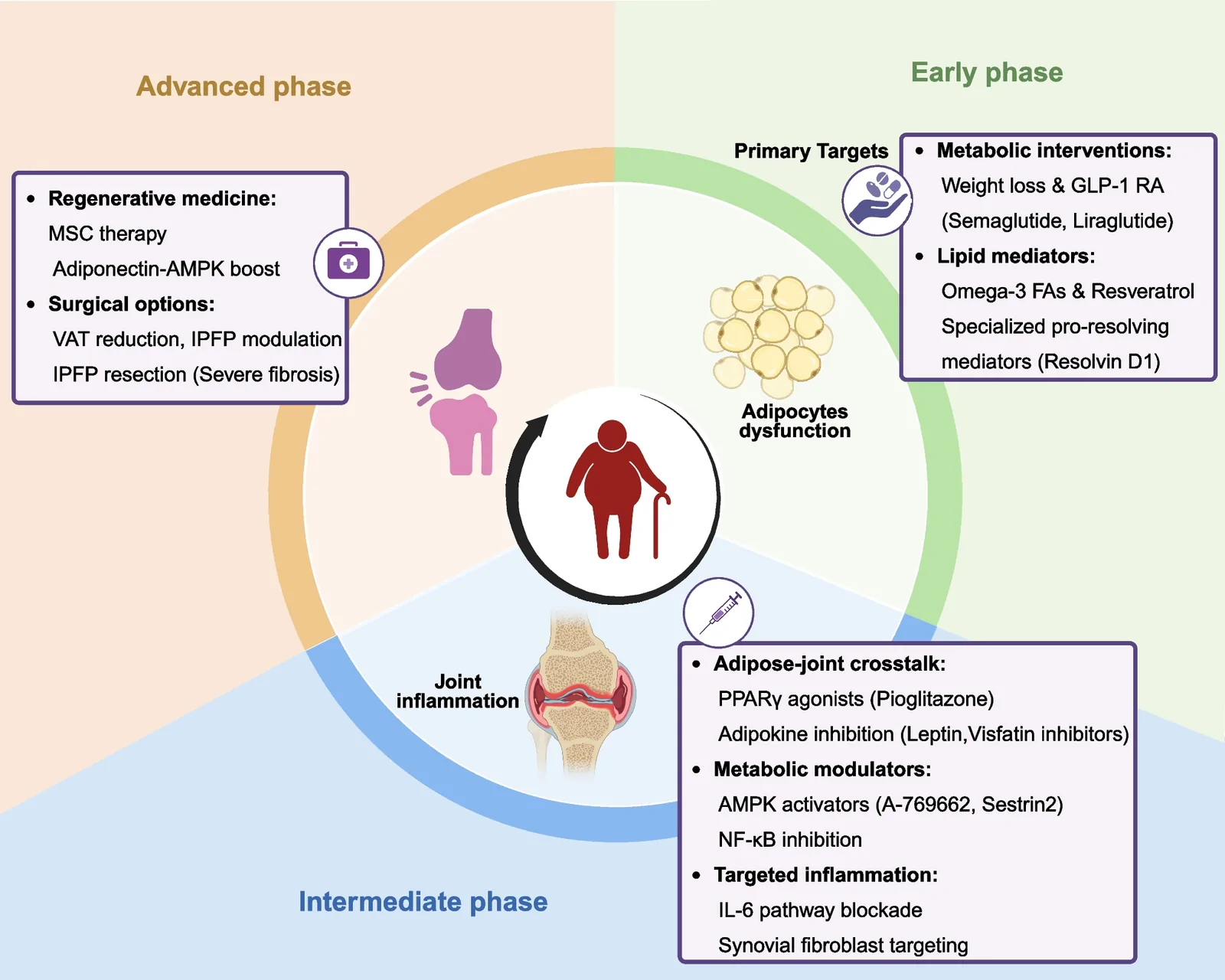
Phase-specific therapeutic strategies for obesity-induced OA. The circular diagram maps the progression of obesity-induced osteoarthritis (OA) and corresponding therapeutic windows across three disease phases, centred on the patient with obesity and OA. Early phase (green): the optimal metabolic intervention window, before irreversible joint damage. Treatments include weight loss, glucagon-like peptide-1 receptor agonists (GLP-1 RA; semaglutide, tirzepatide), resveratrol (TLR4 inhibition), omega-3 fatty acids, and specialised pro-resolving mediators (resolvin D1). Intermediate phase (blue): established joint inflammation with ongoing metabolic dysfunction. Treatments include peroxisome PPARγ agonists (pioglitazone), cytokine inhibitors, adipokine pathway modulators (leptin and visfatin targeting), AMPK activators, NF-κB regulators, IL-6 pathway blockade, and synovial fibroblast-targeted agents. Advanced phase (orange): severe structural damage and metabolic dysfunction. Treatments include mesenchymal stem cell (MSC) therapy with adiponectin-AMPK pathway enhancement, VAT reduction, IPFP modulation, and IPFP resection for severe fibrosis. (Created in BioRender. Z, Y. (2026) https://BioRender.com/w44psyz)

Obesity-induced OA represents a distinct disease entity with identifiable metabolic drivers that create opportunities for therapeutic development. Mapping the temporal progression model with targeted therapeutic approaches identifies optimal intervention windows, as shown in Fig. 4.

### Early Phase: Metabolic Intervention Window

The early phase represents the optimal therapeutic intervention window, where targeted metabolic therapies can effectively prevent progression to irreversible joint damage. Key therapeutic strategies include systemic metabolic modulators, specialized lipid mediators, and cellular metabolic reprogramming.

Weight loss through lifestyle interventions including caloric restriction and structured exercise programs reduce systemic inflammation through decreased VAT mass and improved adipokine profiles, with weight loss of 10% body weight significantly reducing serum leptin levels and improving knee OA symptoms [90]. GLP-1 receptor agonists demonstrate direct anti-inflammatory effects on joint tissues. Liraglutide treatment significantly decreases inflammatory mediators in chondrocytes [91]. Early clinical trials show that semaglutide provides significant pain reduction in patients with obesity and knee OA [89].

Specialized lipid mediators and metabolic modulators offer additional therapeutic avenues. Omega-3 fatty acid supplementation reduces inflammatory eicosanoid production while promoting synthesis of specialized pro-resolving mediators [92], with resolvin D1 demonstrating potent chondroprotective effects [93]. Resveratrol shows promise through dual mechanisms of reducing systemic inflammation and inhibiting TLR4 signalling in cartilage [94]. Gut microbiome manipulation using prebiotics like oligofructose represents a novel approach [95]. These interventions target upstream drivers before inflammatory cascades become self-sustaining.

### Intermediate Phase: Multi-Target Intervention

The intermediate phase necessitates multi-target interventions addressing adipose and joint tissue dysfunction combined with immune reprogramming strategies to prevent progression to irreversible tissue damage.

Metabolic pathway modulators address cellular dysfunction at critical control points. NF-κB and AMPK represent key intervention targets controlling inflammatory responses and cellular metabolism (Section "Molecular signalling convergence in joint tissues"). AMPK activators like A-769662 promote PGC-1α expression and rescue deficient mitochondrial biogenesis in OA chondrocytes through SIRT1 signalling [96]. Sestrin2 overexpression ameliorates OA pain via AMPK/PGC-1α-mediated mitochondrial biogenesis restoration while suppressing neuroinflammation [97].

Targeting adipose-joint crosstalk interrupts pathological communication networks that drive disease progression. PPARγ agonists like pioglitazone demonstrate dual benefits by restoring healthy adipocyte function and directly protecting cartilage [41], with recent studies revealing PPARγ activation alleviates OA through inhibiting pyroptosis and enhancing mitochondrial function [98]. Anti-leptin strategies including neutralizing antibodies and receptor blockade show efficacy in preclinical models [99]. Cytokine inhibition focuses on IL-6 pathway blockade to reduce cartilage damage, synovial inflammation, and subchondral bone pathology [100]. Visfatin inhibitors like APO866 demonstrate efficacy in reducing inflammatory mediator production [101]. Targeting synovial fibroblasts emerges as a high-priority strategy given their central role in perpetuating inflammatory cascades. Multi-target strategies address the networked nature of obesity-induced OA rather than isolated mediators.

### Advanced Phase: Comprehensive Disease-Modifying Strategies

The advanced phase requires comprehensive disease-modifying strategies, potentially including tissue engineering approaches and surgical interventions, combined with metabolic management. Therapeutic focus shifts to adipose tissue-targeted approaches for depot-specific interventions and regenerative medicine designed to overcome the hostile metabolic environment.

Adipose tissue-targeted approaches recognize heterogeneous contributions of different adipose compartments. VAT reduction through dietary intervention and exercise provides systemic anti-inflammatory effects [102]. IPFP management requires nuanced approaches, though severe cases marked by IPFP fibrosis and synovitis may require selective surgical resection [88]. Clinical translation priorities include developing standardized imaging-based protocols for measuring VAT, SCAT, and IPFP volumes to guide targeted interventions.

Regenerative medicine approaches face unique challenges in obesity-induced OA due to the hostile metabolic environment that impairs cellular function. Mesenchymal stem cell (MSC)-based therapies offer promising prospects, with adiponectin-mediated AMPK pathway activation emerging as a strategy for enhancing adipose-derived stem cell function [103]. Clinical evidence demonstrates that intra-articular MSC injection provides significant improvements in pain and function scores [104]. The inflammatory OA environment reduces MSC survival and therapeutic effectiveness, requiring approaches to enhance cellular resilience before implantation [105]. Advanced phase strategies must address both the consequences of long-term metabolic dysfunction and optimize the tissue environment for regenerative interventions.

### From Biomarkers to Clinical Practice

Translating mechanistic insights into clinical practice requires validated biomarkers that enable early disease detection, treatment monitoring, and prognostic stratification in obesity-induced OA. Serum adipokine profiles, particularly leptin, adiponectin, visfatin, and resistin, combined with inflammatory markers (CRP, IL-6) and lipid assessments (ox-LDL, HDL functionality), show promise for identifying at-risk obese individuals [106]. Recent evidence demonstrates that leptin and complement factor D serve as key mediators linking adipose tissue dysfunction to OA severity, suggesting these molecules as particularly informative biomarkers [107]. Metabolic phenotyping can distinguish visceral adipose tissue-driven systemic inflammation from infrapatellar fat pad-driven local pathology, enabling targeted intervention selection [46]. Synovial fluid cytokine profiling may predict response to targeted anti-inflammatory therapies, while monitoring established biomarkers during metabolic interventions provides objective evidence of therapeutic efficacy [108].

Implementing biomarker-guided precision medicine requires integrated care models coordinating expertise across obesity medicine, rheumatology, and orthopedic surgery. Recognizing weight management and metabolic optimization become primary therapeutic goals alongside OA symptom control [87]. GLP-1 receptor agonists exemplify this translational opportunity, as emerging evidence demonstrates their capacity to interrupt inflammatory cascades and modulate adipokine-mediated joint pathology [109]. Addressing the convergent epidemics of obesity and osteoarthritis requires recognizing their bidirectional mechanistic relationship, where metabolic dysfunction and systemic inflammation drive disease pathogenesis [110]. Effective management targeting upstream metabolic pathways, including weight reduction, metabolic modulation, and anti-inflammatory interventions, is needed rather than relying solely on symptomatic relief.

### Clinical Trial Strategies

Advancing evidence-based management of obesity-induced OA requires designed clinical trials addressing critical knowledge gaps across the disease spectrum. Prevention trials should evaluate metabolic interventions including GLP-1 receptor agonists, dietary modifications, and structured exercise programs in high-risk obese individuals without radiographic OA. Recent systematic reviews of metabolic therapies provide foundational evidence, yet prospective trials specifically designed to assess OA prevention in metabolically high-risk populations remain critically needed [111].

Combination therapy trials evaluating synergistic effects of metabolic interventions paired with targeted anti-inflammatory therapies could identify optimal treatment algorithms. Given evidence that obesity and joint injury exert synergistic effects on cartilage degradation, post-traumatic OA prevention trials should specifically evaluate whether metabolic optimization immediately following injury can reduce subsequent disease development [112]. Recognizing that obesity-induced OA pain mediated by adipose tissue dysfunction suggests trials should assess pain outcomes using multidimensional tools capturing both nociceptive and neuropathic components [108]. Addressing fibrotic processes in obesity-related OA represents an emerging therapeutic frontier requiring mechanistic trials targeting extracellular matrix remodelling pathways [113].

## Future Directions and Conclusion

This comprehensive review elucidates the intricate pathophysiological mechanisms underlying obesity-induced OA. The evidence presented establishes obesity-induced OA as a complex disorder driven by dysfunctional adipose-joint communication networks, systemic metabolic perturbations, and coordinated cellular reprogramming across multiple tissue compartments.

Adipose tissue emerges as the fundamental pathological driver, undergoing dysfunction across multiple anatomical depots including subcutaneous, visceral, intramuscular, and critically, periarticular locations such as the infrapatellar fat pad. These dysfunctional adipose tissues demonstrate characteristic cellular remodelling featuring adipocyte hypertrophy and hyperplasia, resulting in aberrant secretion of pro-inflammatory adipokines (leptin, resistin, visfatin, chemerin) while reducing protective anti-inflammatory mediators like adiponectin. Dysregulated adipokines directly stimulate chondrocytes to increase production of matrix-degrading enzymes (MMPs, ADAMTS) and pro-inflammatory cytokines through NF-κB and MAPK signalling pathway activation. Adipose tissue demonstrates substantial infiltration of pro-inflammatory immune populations, including pro-inflammatory macrophages, activated neutrophils, Th1 and Th17 cells, while protective regulatory T cell populations decline significantly. These immunological shifts extend beyond local tissue effects to fundamentally alter joint microenvironments. Adipokine-activated synovial fibroblasts amplify these inflammatory responses while recruiting additional immune cells through enhanced chemokine secretion. Joint tissue transformation creates conditions that favour progressive joint degeneration over tissue restoration. The cellular heterogeneity revealed through single-cell analyses enables precision medicine approaches.

The mechanistic insights presented demand a shift from symptomatic management to precision, mechanism-based interventions. Future combination therapeutic approaches should simultaneously address multiple pathological axes. Firstly, precision multi-target protocols can optimize therapeutic selection through biomarker-guided approaches. Individual patient profiling using adipokine panels, inflammatory markers, and cellular composition analysis will determine optimal combinations of metabolic, inflammatory, and regenerative interventions. Secondly, temporal optimization of combination therapy timing based on disease progression stage and cellular state transitions will maximize therapeutic efficacy. This calls for establishing optimal dosing and timing protocols based on disease progression patterns. Thirdly, multi-target therapeutic strategies show considerable promise across several domains: anti-cytokine approaches, particularly IL-6 and leptin pathway inhibition, address core inflammatory drivers; metabolic modulators including PPARγ agonists and glycolysis inhibitors target upstream metabolic dysfunction. Finally, regenerative MSC therapy combined with systemic metabolic improvement offers long-term therapeutic potential. Ex vivo MSC engineering through mTOR inhibition and AMPK activation enhances cellular stress resistance independent of systemic adipokine delivery challenges.

With over 4 billion people projected to be affected by obesity by 2035, the obesity-induced OA epidemic represents an urgent public health priority. By recognizing obesity-induced OA as a treatable metabolic disorder and targeting the adipose-joint axis at multiple levels, we can transform this condition into a preventable and reversible disease.

## Key References

- Collins KH, Lenz KL, Pollitt EN, Ferguson D, Hutson I, Springer LE, et al. Adipose tissue is a critical regulator of osteoarthritis. Proc Natl Acad Sci U S A. 2021;118(1):e2021096118. https://doi.org/10.1073/pnas.2021096118.
  - This paper provides direct causal evidence that adipose tissue, independent of mechanical loading, drives OA pathology in mice.
- Wijesinghe SN, Badoume A, Nanus DE, Sharma-Oates A, Farah H, Certo M, et al. Obesity defined molecular endotypes in the synovium of patients with osteoarthritis provides a rationale for therapeutic targeting of fibroblast subsets. Clin Transl Med. 2023;13(4):e1232. https://doi.org/10.1002/ctm2.1232.
  - It provides key single-cell evidence for obesity-associated synovial fibroblast heterogeneity in OA.
- Bliddal H, Bays H, Czernichow S, Hemmingsson JU, Hjelmesæth J, Morville TH, et al. Once-Weekly Semaglutide in Persons with Obesity and Knee Osteoarthritis. N Engl J Med. 2024;391(17):1573–83. https://doi.org/10.1056/NEJMoa2403664.
  - This clinical trial demonstrates that GLP-1 receptor agonist therapy benefits patients with obesity and knee OA.
- Binvignat M, Sellam J, Berenbaum F, Felson DT. The role of obesity and adipose tissue dysfunction in osteoarthritis pain. Nat Rev Rheumatol. 2024;20(9):565–84.https://doi.org/10.1038/s41584-024-01143-3.
  - It comprehensively reviews the mechanisms linking obesity, adipose tissue dysfunction, and OA pain.
- Tang S, Zhang C, Oo WM, Fu K, Risberg MA, Bierma-Zeinstra SM, et al. Osteoarthritis. Nat Rev Dis Primers. 2025;11(1):10.https://doi.org/10.1038/s41572-025-00594-6.
  - It provides an authoritative overview of OA, including the role of obesity as a major modifiable risk factor.

## Data Availability

No datasets were generated or analysed during the current study.

## Abbreviations

ADAMTS: A Disintegrin and Metalloproteinase with Thrombospondin Motifs
AMPK: AMP-Activated Protein Kinase
AP-1: Activator Protein 1
APRIL: A Proliferation-Inducing Ligand
BAFF: B Cell Activating Factor
BMAT: Bone Marrow Adipose Tissue
CCL: C–C Motif Chemokine Ligand
COX-2: Cyclooxygenase-2
CRP: C-Reactive Protein
CXCL: C-X-C Motif Chemokine Ligand
DAMP: Damage-Associated Molecular Pattern
DPP4: Dipeptidyl Peptidase 4
ECM: Extracellular Matrix
ERK: Extracellular Signal-Regulated Kinase
FFA: Free Fatty Acid
GAS6: Growth Arrest-Specific Protein 6
GLP-1: Glucagon-Like Peptide-1
HDL-c: High-Density Lipoprotein Cholesterol
HTC: Hypertrophic Chondrocyte
ICAM-1: Intercellular Adhesion Molecule 1
IFN-γ: Interferon Gamma
IL: Interleukin
IMAT: Intramuscular Adipose Tissue
IPFP: Infrapatellar Fat Pad
JAK2: Janus Kinase 2
JNK: C-Jun N-Terminal Kinase
LOX-1: Lectin-Like Oxidized LDL Receptor-1
LOXL3: Lysyl Oxidase-Like 3
MAPK: Mitogen-Activated Protein Kinase
MCP-1: Monocyte Chemoattractant Protein-1
MMP: Matrix Metalloproteinase
MSC: Mesenchymal Stem Cell
mTOR: Mechanistic Target of Rapamycin
mTORC1: mTOR Complex 1
NF-κB: Nuclear Factor Kappa B
NLRP3: NLR Family Pyrin Domain Containing 3
NR3C1: Nuclear Receptor Subfamily 3 Group C Member 1
OA: Osteoarthritis
OSM: Oncostatin M
ox-LDL: Oxidized Low-Density Lipoprotein
PGC-1α: Peroxisome Proliferator-Activated Receptor Gamma Coactivator 1-Alpha
PGD2: Prostaglandin D2
PGE2: Prostaglandin E2
PI3K: Phosphoinositide 3-Kinase
PPARγ: Peroxisome Proliferator-Activated Receptor Gamma
RANKL: Receptor Activator of Nuclear Factor Kappa-B Ligand
ROS: Reactive Oxygen Species
SCAT: Subcutaneous Adipose Tissue
SIRT1: Sirtuin 1
STAT3: Signal Transducer and Activator of Transcription 3
Th1: T Helper Type 1
Th17: T Helper Type 17
TIMP: Tissue Inhibitor of Matrix Metalloproteinases
TLR: Toll-Like Receptor
TNF-α: Tumor Necrosis Factor Alpha
TREG: Regulatory T Cell
TRPV1: Transient Receptor Potential Vanilloid 1
VAT: Visceral Adipose Tissue
VCAM-1: Vascular Cell Adhesion Molecule 1
VEGF: Vascular Endothelial Growth Factor

## Declarations

### Literature Search and Study Selection

We systematically searched PubMed databases for publications from January 2010 through December 2025 combining obesity and osteoarthritis terms, supplemented by targeted searches for specific mechanisms and advanced methodologies. Studies were prioritized based on mechanistic depth, methodological innovation (single-cell sequencing, spatial transcriptomics), and therapeutic relevance, emphasizing publications providing molecular or cellular insights into obesity-induced OA pathogenesis.
